# Quantification of compensatory processes of postnatal hypoxia in newborn piglets applying short-term nonlinear dynamics analysis

**DOI:** 10.1186/1475-925X-10-88

**Published:** 2011-10-03

**Authors:** Steffen Schulz, Sina Reulecke, Michael Eiselt, Karin Schwab, Herbert Witte, Bernd Walter, Reinhard Bauer, Andreas Voss

**Affiliations:** 1 Department of Medical Engineering and Biotechnology, University of Applied Sciences, Jena, Germany; 2 Institute of Medical Statistics, Computer Sciences and Documentation, Friedrich-Schiller-University, Jena, Germany; 3 Institute of Molecular Cell Biology, University Hospital, Friedrich-Schiller-University, Jena, Germany

## Abstract

**Background:**

Newborn mammals suffering from moderate hypoxia during or after birth are able to compensate a transitory lack of oxygen by adapting their vital functions. Exposure to hypoxia leads to an increase in the sympathetic tone causing cardio-respiratory response, peripheral vasoconstriction and vasodilatation in privileged organs like the heart and brain. However, there is only limited information available about the time and intensity changes of the underlying complex processes controlled by the autonomic nervous system.

**Methods:**

In this study an animal model involving seven piglets was used to examine an induced state of circulatory redistribution caused by moderate oxygen deficit. In addition to the main focus on the complex dynamics occurring during sustained normocapnic hypoxia, the development of autonomic regulation after induced reoxygenation had been analysed. For this purpose, we first introduced a new algorithm to prove stationary conditions in short-term time series. Then we investigated a multitude of indices from heart rate and blood pressure variability and from bivariate interactions, also analysing respiration signals, to quantify the complexity of vegetative oscillations influenced by hypoxia.

**Results:**

The results demonstrated that normocapnic hypoxia causes an initial increase in cardiovascular complexity and variability, which decreases during moderate hypoxia lasting one hour (p < 0.004). After reoxygenation, cardiovascular complexity parameters returned to pre-hypoxic values (p < 0.003), however not respiratory-related complexity parameters.

**Conclusions:**

In conclusion, indices from linear and nonlinear dynamics reflect considerable temporal changes of complexity in autonomous cardio-respiratory regulation due to normocapnic hypoxia shortly after birth. These findings might be suitable for non-invasive clinical monitoring of hypoxia-induced changes of autonomic regulation in newborn humans.

## Background

The adaptation to extrauterine life, also referred to as transition, involves functional modifications in virtually every organ and bodily system. The most crucial event is the conversion of the fluid-filled lungs into a hollow organ distended with air and capable of gaseous exchange sufficient to warrant extrauterine life. Although the majority of newly born humans establish normal respiratory and circulatory function, 1-2% may run into difficulties due to a disturbance to the normal adaptive processes required for a smooth transition from intrauterine to extrauterine life [1]. Under such circumstances, sustained systemic hypoxia (for review see [2]) is a common consequence and may lead to fatality or severe lifelong disabilities in case of a progressive lack of oxygen. However, moderate systemic hypoxia of a lower degree is compensated by the neonate during a prolonged time period [3]. This is due to a well-established and coordinated neuroendocrine response to systemic hypoxia which functions at birth and is controlled mainly by complex autonomic processing. However, there is a lack of knowledge about the time- and intensity-related alterations of autonomic activity in cardio-respiratory control under hypoxic conditions during the newborn's first period of life. The newborn piglet achieves a degree of maturity at birth similar to that of humans with regard to cardiovascular regulation [4]. Therefore, this species have been considered to be an excellent sub-primate laboratory model for comparison studies with human infants. Previous studies using a swine model have shown that hypoxia causes alterations in the autonomic system. Zwiener [5] investigated deterministic-chaotic properties that changed after exposure to hypoxia. Gootman [3] determined that hypoxia leads to increased sympathetic activity and to augmented respiratory modulation, but that vagal innervations are inhibited. Evaluations of heart rate variability changes were performed by Sica & Zhao [6] with a focus on linear methods as spectral features. In these studies mainly linear measures from time- and frequency domains [3,6-9] were applied.

Nonlinear methods were considered only in a few studies. Zwiener [5] investigated hypoxia, analysing signals from the autonomic nerve system using the correlation dimension, Lyapunov exponent and phase space plots. However, these classical nonlinear methods suffer from the dimensionality curse and require a large number of data points in the time series to reliably estimate the nonlinear features. Unfortunately, this prevents the analysis of short-term time series and the interactions between different signals due to the complex autonomic regulation. On the other hand nonlinear measures based on entropy estimation as Approximate Entropy (ApEn) or Sample Entropy (SampEn) were able to quantify heart rate irregularity with short segments during episodes of mechanical ventilation and acute anoxia in rats[10]. Though, to determine the nonlinear interplay of various physiological control loops, a multivariate approach based on a combination of different linear and nonlinear parameters is required [11,12]. Short-term dynamics due to sustained hypoxia have not been investigated in very young spontaneously breathing piglets thus far. Further, there is only limited information about the complex interactions between cardiovascular and respiratory signals that are altered during the dynamic development from normoxia to hypoxia and subsequently during reoxygenation.

The aim of this study was to characterize physiological states before, during and after hypoxia exposure to spontaneously-breathing piglets, contributing to the understanding of compensation processes in newborns due to postnatal hypoxia applying short-term nonlinear dynamics analysis. Moreover, the temporal and complex development of the autonomic regulation had to be explicitly considered between the phases inside states according to normoxia, hypoxia and reoxygenation. For this purpose, a new algorithm for testing stationarity in a short-term time series was introduced. Linear and especially nonlinear methods were then applied to stationary time series (cardiovascular and respiratory) to investigate the complexity of physiological dynamics inherent in the autonomous system [13]. Several of these nonlinear dynamics have been proven to be of diagnostic relevance or have contributed to risk stratification [12]. We hypothesize that linear and especially nonlinear short-term indices, when applied to cardiovascular and respiratory signals, can reveal alterations and changes in complexity during the autonomic regulation process of piglets due to their adaptation to hypoxia. Hence, a greater comprehension of temporal and intensity characteristics of autonomic tone might be possible with regard to the adaptive capacity of newborns during a reduced and re-established oxygen supply. In addition, findings from this study could be useful in the paediatric field for the purpose of recognizing and monitoring hypoxic effects in human newborns.

## Methods

### Animals and experimental procedure

Animal data were used from seven piglets enrolled in a previous study to estimate the effect of artificial ventilation on regional blood flow and on cardiovascular regulation in newborns [14]. The study was approved by the Committee of the Thuringian State Government for Animal Research. The animals were managed in accordance with the guidelines of the American Physiological Society. In the previous study these seven newborn piglets (2-3 days old, 1.71 ± 0.15 kg b.w.) served as a reference group, breathing spontaneously under normoxia and normocapnic hypoxia. They were initially anesthetized with 2.5% isoflurane in nitrous oxide and were given oxygen via a mask. They were maintained throughout the surgical procedure using 1.3% isoflurane in order to record cardio-respiratory signals. During the first 60 minutes, animals stayed under resting conditions (normoxia). After monitoring the resting state, the inspired oxygen fraction was reduced from 0.3 to about 0.1 via an appropriate exchange with nitrogen, causing a normocapnic hypoxia for approximately 60 min. Finally the gas mixture was re-established to record recovery over a time period of 30 min (reoxygenation).

### Data acquisition and pre-processing

High resolution ECG (2048 Hz sampling frequency), synchronized continuously blood pressure and respiration signals were recorded from all seven subjects. Recording time varied from 137 to 191 minutes. ECG was measured by standard limb leads using stainless steel needle electrodes (HSE EKA - Puls IC, Hugo Sachs Elektronik K.G., Germany), while respiration waves were recorded by impedance plethysmography using sticking electrodes on both sides of the chest wall (Cardio-Respiratorischer Monitor/Apnocard 300, Mechanische Werkstätten Radeberg, Germany). Aside, one catheter was advanced through an umbilical artery into the abdominal aorta to record arterial blood pressure (P23Db, Statham Instruments Inc., Hato Rey, Puerto Rico). Physiological parameters were recorded on a multichannel polygraph (MT95K2, Astro-Med, W. Warwick, U.S.A.) and stored on hard-disk. For analysis, a number of factors that could affect the obtained results via linear and nonlinear methods had to be considered, for example the degree of stationarity, superimposed noise and signal pre-processing (filtering) [12]. Therefore, it became necessary to set up pre-selection criteria. Due to the bi- and multivariate approaches which had been applied in our analyses, it was necessary that adequate processable signals (namely ECG, blood pressure and respiration) with sufficient signal quality and without artefacts were simultaneously available for further processing. Because of the previous study design [14], the aforementioned requirements for signal quality were not present all the time during the investigation. An additional criterion was stationarity due to the fact that some of the applied methods (namely frequency domain analysis and cross conditional entropy) need stationarity conditions.

**Figure 1:**
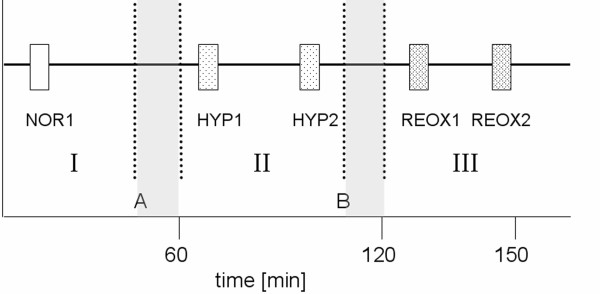
**Schematic sketch of phase extraction**. Stationary signals were extracted from all time series (BBI, SYS, DIA and RESP) from the normoxia (I), hypoxia (II) and reoxygenation (III) periods. Phases NOR1, HYP1, HYP2, REOX1 and REOX2 were chosen for analyses. A: start of hypoxia; B: end of hypoxia; grey boxes: non-stationary transition phases.

**Figure 2:**
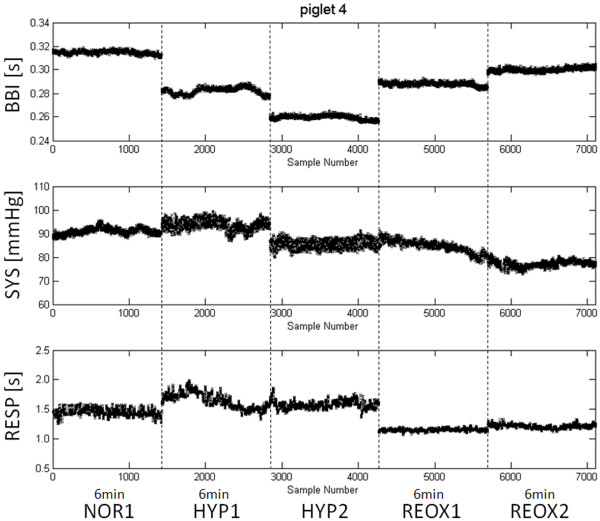
**Representative time series for study analysis**. Parallel stationary time series (6 min) of one piglet's beat-to-beat intervals (BBI), systolic blood pressure (SYS) and respiratory intervals (RESP) for the five analysed sections: normoxia (NOR1), early hypoxia (HYP1), late hypoxia (HYP2), early reoxygenation (REOX1) and late reoxygenation (REOX2).

Stationarity requires that statistical properties such as mean and standard deviation of the investigated time series remain the same throughout the investigated time period. Therefore, we applied a test where the extracted time segments fulfilled our pre-selection criteria (involving stationarity) in all piglets. Subsequently, the dynamic behaviour before, during and after hypoxia was investigated to analyse the changes in autonomic regulation. For this purpose, two sections, each six minutes in length, were selected from raw data records for the states of hypoxia and reoxygenation (Figure 1). One six-minute phase from the beginning of normoxia (NOR1) was used for subsequent comparison with the early hypoxia- (HYP1), late hypoxia- (HYP2), early reoxygenation- (REOX1) and late reoxygenation- (REOX2) phases (Figure 2).

Heart rate time series (tachogram) consisting of successive beat-to-beat intervals (*BBI*) as well as systolic (*SYS*) and diastolic (*DIA*) blood pressure values were extracted from data records. Then these time series were filtered by applying an adaptive variance estimation algorithm to remove and interpolate ventricular premature beats and artefacts (e.g. movement, electrode noise and extraordinary peaks) to obtain a normal-to-normal beat time series (NN) [15]. The phase of respiration was calculated to generate the interval times between consecutive breathing cycles (*RESP*). For the purpose of interaction analyses all four signals were resampled with a frequency of 4 Hz to obtain synchronised time series.

### The extraction of stationary phases

Algorithm:

1. Dividing the entire *BBI *time series of NOR, HYP and REOX into two equal parts.

2. Calculation of the:

a. mean value *meanBBI6 min *[ms],

b. standard deviation *sdBBI6 min *[ms] and

c. variation ratio $vrBBI6min=\frac{sdBBI6\text{min}}{meanBBI6\text{mi}{\text{n}}^{3}}\left[\text{1}∕\text{m}{\text{s}}^{\text{2}}\right]$

for *S *overlapping subsequences (shift = 1 minute) of 6 minutes window length.

3. Extracting the phase with the smallest *vrBBI6 min*.

### Test of stationarity

The stationarity of each calculated phase was subsequently controlled using the following algorithm:

1. Calculations of global mean (*global_mean*) and global standard deviation (*global_std*) from the entire time series.

2. Calculation of two local mean (*local_mean*) and local standard deviation (*local_std*) values for the first and second halves of the extracted stationary phase.

3. Computation of the absolute differences *d_mean *and *d_std *between the global and local value each for both *mean *and *std*.

(1) d_mean(i)=abs(global_mean-local_mean(i))

(2) d_std(i)=abs(global_std-local_std(i));i=1,2

4. Determination of the deviation percentage between the two differences for *mean *and *std *values respectively to quantify the degree of stationarity of the extracted phase.

(3) dev_mean[%]=abs(1-(d_mean(1)∕d_mean(2)))

(4) dev_std[%]=abs(1-(d_std(1)∕d_std(2)))

The results of stationarity analysis showed that averaged *local_mean *differed less than 1% while *local_std *changed to maximally 5%. Due to these marginal deviations, the extracted phases could be evaluated as stationary and were thus used for analysis.

### Analysing autonomic regulation

Heart rate variability (*HRV*) and blood pressure variability (*BPV*) were quantified by well established indices of time- and frequency domains [16] and nonlinear dynamics [12], calculated from univariate cardiovascular and respiratory signals (*BBI, SYS, DIA *and *RESP*). For bivariate analyses, well-established and validated nonlinear methods, that investigate pair-wise interactions between cardiovascular and respiratory signals, were also applied. In addition, all possible combinations of cardiovascular- and cardio-respiratory interactions were analysed (i.e. *BBI-SYS, BBI-DIA, BBI-RESP*, *SYS-DIA, SYS-RESP and DIA-RESP*).

### Time and frequency domains

Using the time domain, the following standard indices were calculated:

• *meanNN *- the mean value of the NN-intervals [ms];

• *sdNN *- standard deviation of the NN-intervals [ms].

To estimate the power spectra of the time series, Fast Fourier Transform with a Blackman Harris window function (to avoid leakage effects) was applied to every complete phase. Frequency domain indices were adapted to temporal and spectral features of neonatal cardiovascular signals [17]: very low frequency power (0.005 - 0.04 Hz), low frequency power (0.04 - 0.25 Hz) and high frequency power (0.25 - 0.85 Hz).

### Symbolic Dynamics

The analysis of Symbolic Dynamics (*SD*) has been proven to be adequate for the investigation of complex systems and can describe nonlinear aspects within short- and long-term time series [13,18]. Symbolic dynamics are based upon a coarse-graining of a system's dynamics. The well-established algorithm of *SD *is performed as follows: to classify dynamic changes within the time series they were first transformed into a symbol sequence of four symbols using the alphabet *A={0,1,2,3}*. Three successive symbols (*k *= 3; k - word length) were used to characterise symbol strings whereby 64 different word types (bins) were obtained (000, 001, ..., 333) according to the transformation [19]. The resulting histogram contains the probability distribution of each single word type within a word sequence. The following indices from this probability distribution were estimated for *BBI, SYS and DIA*:

• *wpsum02 *- a relative portion (sum/total) of words consisting of only the symbols '0' and '2' to measure decreased *HRV *[%]

• *wd_renyi1 *- Renyi entropy of the word type distribution with *α *= 0.25 as a measure of complexity [bit].

The factor α = 0.25 [13] determines the probability distribution *p_i _*of words in the histogram. If 0<*α*, words with small probabilities will mainly determine the value of *H_renyi_(*α).

### Joint symbolic dynamics

To characterize the nonlinear interactions of cardiovascular- and cardio-respiratory interdependencies the method of Joint Symbolic Dynamics (*JSD*) was used. This method is based on the analysis of coarse-grained dynamic processes by means of symbols [13,20,21]. Here cardiovascular- (*BBI-SYS*) and cardio-respiratory (*BBI-RESP*) time series were transformed into symbol sequences *s *of different words according to the transformation rules. Only a short alphabet A={0,1} was used for the symbol sequences whereby increasing values were coded as '1' and decreasing- and unchanged values were coded as '0', respectively. Therefore, short patterns (words of length 3) were formed. From each word type (*k = 64*) the normalized probability occurrences were estimated (*JSD-JSD64 *) using an *8 × 8 *word distribution density matrix (rows - *BBI*, columns - *SYS, RESP*, respectively). The advantages of *JSD *are that it considers all types of interval changes and that a rough assessment of the overall cardiovascular- and cardio-respiratory regulation of short-term interactions is obtained. The following indices were estimated:

• *BBI_SYS_JSD *- normalised probability occurrence of specific word types of heart rate- and systolic blood pressure time series [‰];

• *BBI_RESP_JSD *- normalised probability occurrence of specific word types of heart rate- and inspiratory cycle time series [‰].

The interaction of heart rate time series and diastolic blood pressure time series as well as systolic and diastolic blood pressure time series were not performed due to *JSD *is not validated for these interactions so far.

### Poincaré plot analysis

The Poincaré Plot Analysis (*PPA*) is based on a technique from non-linear dynamics and represents the nature of time series fluctuations and can be utilized for determining heart beat dynamics with trends [22,23]. The Poincaré plots are two-dimensional graphical representations of each value of either *BBI, SYS, DIA *or *RESP *time series plotted against the subsequent value. The Poincaré plot typically illustrates an elongated cloud of points oriented along the line of identity. For the purpose of graphical illustration, an ellipse based on the shape of the point cloud can be drawn in the plot, whereas the centre of the ellipse represents the mean value. The Poincare' plot is based on the notion of different temporal effects due to changes in the vagal and sympathetic modulation of vegetative parameters, without requirement of stationarity in the time series [24]. In general, three indices are calculated based on the Poincaré plots [22,25,26]. In this study we calculated the two standard deviations, namely *SD1 *(the ellipse's minor axis) measuring short-term variability, and *SD2 *(the ellipse's major axis), quantifying long-term variability from the *BBI, SYS, DIA *and *RESP *time series.

### Segmented Poincaré plot analysis

**Figure 3:**
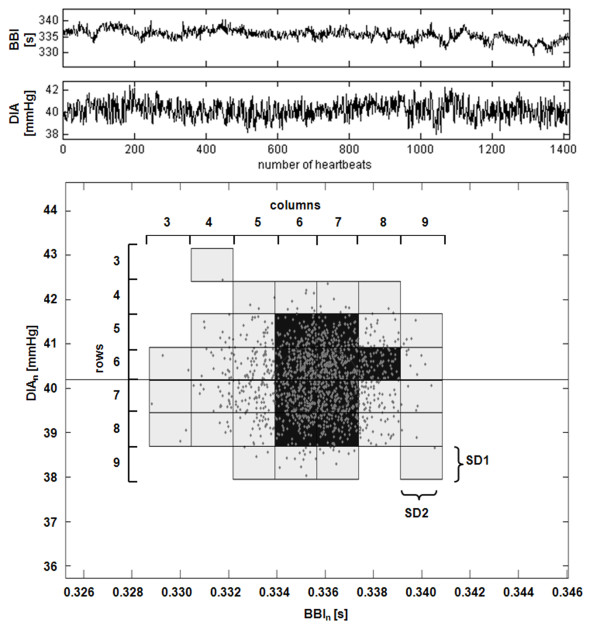
**Illustration of SPPA indices for cardiov ascular interaction**. Graphical representation of indices from segmented Poincaré plot analysis (SPPA) for cardiovascular (BBI and DIA) interaction from one piglet's normoxia phase. Numbered column and row indices are indicated while the width is determined by SD1 (row) and SD2 (column) calculated by standard Poincaré plot analysis (PPA).

The Segmented Poincaré Plot Analysis (*SPPA*) is a quite new enhancement of the *PPA*. Compared to standard *PPA, SPPA *avoids linear correlation and analyses nonlinear features of dynamic systems [27,28]. In addition to the standard *PPA *the graphical representation is divided into sections of 12 symmetrical columns and rows concentrated around the mean value of the scatter plot. The proximity of the particular sections depends on the indices *SD1 *and *SD2 *calculated by the *PPA*. The determined indices represent the percentage of points in each column and row at the rate of all points (Figure 3).

In our study *SPPA *was applied to analyse univariate signals as well as the interactions between two signals. To provide an example, we calculated the following index:

• *BBI_DIA_col5 *- percentage of points in the fifth column of the Poincaré plot of *BBI_n _*vs. synchronous diastolic blood pressure values (*DIA_n_*).

### Cross conditional entropy

Cross Conditional Entropy (*CCE*) provides quantification of the degree of coupling between two signals [29]. Synchronisation occurs when interactive dynamics between two signals are repetitive. Initially the signals are embedded into multiple dimensions. For each dimension the conditional entropy (*CE*) modified from the Shannon entropy is calculated*.CE *is a process of sorting and counting mixed patterns and describes the amount of information included in the sample *y *when the pattern *u *is given. Based on *CE*, one can define the synchronisation index which quantifies the amount of information exchanged between the two signals *u *and *y *[29]. The larger the synchronisation index, the more coupled are the two signals. In our study *CCE *was applied to all possible combinations of cardiovascular- and cardio-respiratory interactions. For example, we calculated the index *SYS_DIA_SI *in order to quantify the coupling between systolic and diastolic blood pressure.

### Statistics

The parametric-paired t-test was applied for the statistical evaluation of all univariate differences in linear and nonlinear parameters between the different time segments of the piglet's data. Moderate univariate significances were considered for values of p < 0.05 (*) and high significances for values of p < 0.01 (**). To verify t-test usage, the Kolmogorov-Smirnov-Test (KS-test) was performed in advance (normal distribution). In addition, adjustments to statistics were applied via multiple testing using the Bonferroni-Holm method [30] to confirm the t-test results (#). Descriptive statistics were used to describe the basic features of the data in terms of mean value (*mean*) and standard deviation (*std*).

## Results

**Figure 4:**
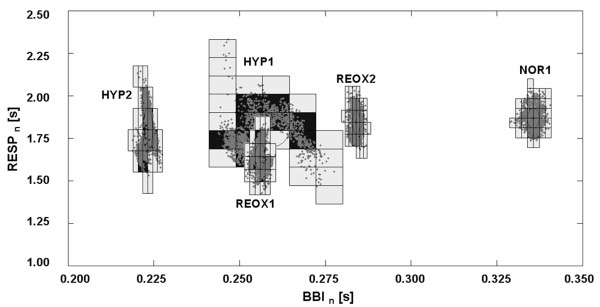
**SPPA graphics of simultaneous cardio-respiratory interactions**. Poincaré plots of synchronised BBI and RESP time series refer to all five phases (NOR1, HYP1, HYP2, REOX1 and REOX2) of the animal experiment using data from one piglet. The grids represent rows and columns filled with data points (approximately inside the 4^th ^till 9^th ^row and column) within the Poincaré cloud. Areas with the highest amount of points are dark highlighted.

The most significant indices, that showed altered dynamics and changes in the complexity of autonomic regulation during hypoxia until the late reoxygenation phase, have been selected. Consequently, the pre-selected indices (Table 1) provide the following results. For analysis purposes, indices from methods *HRV, SD, PPA *and *CCE *showed high significances in discriminating NOR1 from HYP1 and/or HYP2. Regarding the selected indices from these methods, the statistical tests between NOR1 and REOX groups led to various significant trends. Indices from *JSD *and *SPPA *(Figure 4) also revealed high significances for the group tests between NOR1 and REOX1 or REOX2.

**Table 1:** Univariate selected parameters for discrimination between normoxia (NOR1), early hypoxia (HYP1), late hypoxia (HYP2), early reoxygenation (REOX1) and late reoxygenation (REOX2)

		NOR1 **vs**. HYP1	NOR1 **vs**. HYP2	NOR1 **vs**. REOX1	NOR1 **vs**. REOX2	NOR1	HYP1	HYP2	REOX1	REOX2
**method**	***parameter***	**p**	**p**	**P**	**p**	**Mean ± std**	**mean ± std**	**mean ± std**	**mean ± std**	**mean ± std**

HRV	*BBI_meanNN (ms)*	*#	**#	**#	*#	358.02 ± 41.57	296.03 ± 37.77	279.05 ± 40.19	297.19 ± 28.09	295.89 ± 31.83
	*BBI_sdNN (ms)*	*	n.s.	n.s.	n.s.	2.28 ± 1.20	4.49 ± 2.78	2.11 ± 0.69	2.77 ± 1.25	3.25 ± 3.72
	*BBI_LFn (a.u.)*	n.s.	n.s.	n.s.	n.s.	0.41 ± 0.21	0.52 ± 0.19	0.37 ± 0.30	0.38 ± 0.14	0.39 ± 0.20
	*BBI_HFn (a.u.)*	n.s.	n.s.	n.s.	n.s.	0.59 ± 0.21	0.48 ± 0.19	0.63 ± 0.30	0.62 ± 0.14	0.61 ± 0.20
	*BBI_LF/HF (a.u.)*	n.s.	n.s.	n.s.	n.s.	0.93 ± 0.78	1.47 ± 1.17	1.10 ± 1.31	0.68 ± 0.35	0.78 ± 0.55
BPV	*SYS_meanNN (mmHg)*	n.s.	n.s.	n.s.	n.s.	93.95 ± 10.69	99.02 ± 23.89	100.81 ± 27.10	95.75 ± 16.78	90.89 ± 17.03
	*SYS_sdNN (mmHg)*	*	n.s.	n.s.	n.s.	1.75 ± 0.86	3.28 ± 1.84	2.73 ± 1.09	2.10 ± 0.75	2.34 ± 1.93

SD	*BBI_wpsum02 (a.u.,‰)*	n.s.	**#	n.s.	n.s.	99.70 ± 0.03	97.10 ± 4.69	99.77 ± 0.03	99.49 ± 0.50	95.93 ± 9.37
	*SYS_wd_renyi1 (bit)*	**#	**#	n.s.	n.s.	2.34 ± 0.24	2.92 ± 0.32	2.84 ± 0.26	2.53 ± 0.40	2.63 ± 0.41
	*DIA_wd_renyi1 (bit)*	**#	*#	*#	*	2.82 ± 0.51	3.48 ± 0.23	3.43 ± 0.46	3.42 ± 0.22	3.39 ± 0.39
PPA	*BBI_SD1 (ms)*	n.s.	n.s.	n.s.	n.s.	0.99 ± 0.29	0.93 ± 0.26	0.97 ± 0.24	1.06 ± 0.42	1.23 ± 0.72
	*SYS_SD1 (ms)*	**#	**#	*#	n.s.	0.70 ± 0.09	1.05 ± 0.21	1.03 ± 0.26	0.90 ± 0.16	0.91 ± 0.30
	*DIA_SD1 (ms)*	**#	**#	n.s.	*	0.85 ± 0.13	1.42 ± 0.44	1.51 ± 0.60	1.10 ± 0.28	1.16 ± 0.30
SPPA	*RESP_row8 (%)*	n.s.	n.s.	**#	n.s.	12.40 ± 2.29	10.01 ± 4.50	8.86 ± 4.78	8.21 ± 2.15	11.73 ± 2.87
	*BBI_DIA_col5 (%)*	*	n.s.	n.s.	n.s.	13.86 ± 3.24	18.82 ± 4.61	13.87 ± 4.57	13.13 ± 4.34	13.66 ± 3.74
JSD	*BBI_SYS_JSD (a.u.,‰)*	*	*	n.s.	n.s.	15.18 ± 4.48	8.66 ± 6.46	7.72 ± 4.79	13.34 ± 5.15	12.79 ± 4.92
	*BBI_RESP_JSD (a.u.,‰)*	n.s.	n.s.	*	**#	9.40 ± 3.34	5.05 ± 4.43	5.86 ± 5.47	3.99 ± 3.93	3.23 ± 2.25
CCE	*SYS_DIA_SI (a.u.)*	**#	**#	n.s.	n.s.	0.06 ± 0.01	0.11 ± 0.05	0.13 ± 0.07	0.07 ± 0.02	0.09 ± 0.04

(HRV - Heart Rate Variability; BPV - Blood Pressure Variability; SD - Symbolic Dynamics; JSD - Joint Symbolic Dynamics; (S)PPA - (Segmented) Poincaré Plot Analysis; CCE - Cross Conditional Entropy; mean - mean value; std - standard deviation; * - p < 0.05; ** - p < 0.01; n.s. - not significant; # - significant after Bonferroni-Holm adjustment.)

### Linear dynamics

In this context the value of the *HRV *index *BBI_meanNN *decreased from NOR to HYP2 and revealed an increased significance level (p = 0.005). During the process of reoxygenation, the value of *BBI_meanNN *slightly increased, but remained significantly decreased (p = 0.01) in comparison to NOR1. Another time domain parameter, namely *BBI_sdNN*, was significantly (p = 0.04) higher within the HYP1 group compared to NOR1. During the process from HYP2 to REOX2, this index showed a transient trend to values similar to NOR1. Regarding *BPV *analysis, indices showed no significant changes for mean values, but instead an altering progression for blood pressure variance. As a result, the time domain index *sdNN *increased significantly for both systolic and diastolic blood pressure (*SYS*: p = 0.05, *DIA*: p = 0.02) in HYP1 compared to NOR1.

The mean value of the ratio of the powers in the low (*LF*) and the high (*HF*) frequency range, *LF/HF*, from NN-intervals was higher in hypoxia than in normoxia and reoxygenation. However these indices revealed no significant differences caused by relatively high *std*, but *LF *power tend to increase due to hypoxia.

### Nonlinear dynamics

*SD *complexity measure *wpsum02*, a measure for decreased *HRV*, significantly increased (p = 0.005) in HYP2 for *BBI*. Concerning *BPV*, the complexity index *wd_renyi1 *showed high significances for both systolic and diastolic blood pressure for the comparison between NOR1 and HYP1. This parameter lasted high significantly increased for *SYS *and moderate significantly for *DIA *during HYP2. During reoxygenation *SYS_wd_renyi1 *adapted to values similar to NOR1 without any significant differences while *DIA_wd_renyi1 *maintained significantly (p = 0.05) increased. Short-term blood pressure fluctuations were found to be high significantly (p = 0.001) increased during both hypoxia states. This was revealed by the *PPA *index *SD1*. According to *JSD*, the significances of index *BBI_RESP_JSD *revealed an increasing trend (from p = 0.017 to p = 0.003) during reoxygenation. This index describes the percentage of contrary cardio-respiratory interactions which significantly decreased from REOX1 to REOX2. Confirmedly, the univariate *SPPA *index *RESP_row8 *showed a high significance (p = 0.004) for group test NOR1 vs. REOX1. On the contrary, the cardiovascular index *BBI_SYS_JSD *significantly (p = 0.014) declined in HYP1 and HYP2 compared to NOR1. During the investigation of differences caused by hypoxia, significant results were also found using the *CCE *method. In this regard, the synchronisation index *SYS_DIA_SI*, describing the coupling quantity of *DIA *and *SYS *(synchronisation), increased high significantly (p = 0.009) for both hypoxia states.

## Discussion

In this study we analysed the effect of hypoxia on autonomic regulation in newborn piglets by applying linear and nonlinear methods to cardiovascular and respiratory signals. The results indicated that control mechanisms of the autonomic nervous system (*ANS*) in newborn piglets are influenced considerably by exposure to a hypoxic condition as well as to the inversion to normoxia. The results demonstrated that hypoxic conditions lead to an increase in sympathetic activity which decreases during the hypoxia process. This is in accordance with other studies concerning the analysis of autonomic regulation in piglets exposed to hypoxia [3,5,9,14,31-33]. These findings indicate that hypoxia induces time-dependent changes in the cardiovascular and respiratory nervous system. In fact, acute normocapnic hypoxia decreases cardiac vagal tone and increases sympathetic activity resulting in an increased respiration rate and heart rate. After resetting the normoxic supply, cardiovascular and respiratory response diminished as the indices variably changed to conditions similar to those observed before hypoxic exposure.

Regarding linear time domain analyses, *HRV *index *BBI_meanNN *showed changes in autonomic regulation due to hypoxia. The adaptation to the restricted oxygen supply proceeded faster and more strongly while the recovery process proceeded in a slower and rather unexpected way. The time domain index *BBI_sdNN *could only discriminate between NOR1 and HYP1. This result suggests that the piglets regulate with a higher initial variance, but then already regulate to lower variability in terms of adapting to the oxygen deficit. Harris et al. [34] investigated the associations between cardiovascular function and neurological outcome following acute global hypoxia in 20 anaesthetized newborn piglets and found a high degree of variability in cardiovascular function (mean arterial blood pressure and HR) in response to hypoxia. In addition, our study revealed that increased HRV diminished during prolonged hypoxia. This was determined through short-term analyses from early and late time periods during hypoxia. During reoxygenation, *BBI_sdNN *showed a stronger trend towards values that conformed to normoxic conditions. By contrast, the *BBI_meanNN *has not already been regulated to the normoxic level at the end of reoxygenation. Significances revealed by *HRV *indices could only be confirmed for *BBI_meanNN *using the Bonferroni-Holm method. *BPV *time domain analysis revealed in part similar findings to that of *HRV*. The adaptation during reoxygenation to normoxia proceeded faster, probably because of the priority of blood pressure regulation. The standard time domain indices *SYS_meanNN *and *DIA_meanNN *did not change significantly during the entire investigation. This is in accordance with a study by Gootman [3] showing that no significant changes in blood pressure amplitude are characteristic of premature responses to stimulation of baroreceptors and cardiopulmonary receptors. Moreover, they suggest a direct, hypoxia-induced, stimulation of the sympathetic nervous system (HR increase), overriding any baroreceptor inhibition due to the 10% increase in blood pressure.

In addition indices derived from frequency domain revealed no significances in discriminating groups although a shift of *LF *and *HF *components towards higher frequencies was noticed as described by Sica [9], reflecting much higher respiratory and heart rates. Respiration peaks were mostly located at high frequency range between 0.5 and 0.8 Hz associated with vagal control of heart rate [31]. The progress of *HRV *index *LF/HF *indicated to a parasympathetic withdrawal and an ongoing sympathetic predominant activation in autonomic tone during hypoxia (Table 1). These influences diminished after reversal to reoxygenation.

The nonlinear *HRV *index *BBI_wpsum02 *showed an increased complexity of *BBI *at the beginning of hypoxia. Therefore the symbol-coded time series of the early hypoxic *BBI *included more different word types, indicating higher complexity. At the end of the hypoxic state this trend reversed because *HRV *complexity measure *BBI_wpsum02 *significantly increased. Hence, a higher number of words including only symbols "0" and "2" implies lower variability and, as a result, less complexity [35]. Likewise, the entropy index *wd_renyi1 *revealed a higher complexity for both systolic and diastolic blood pressure under hypoxic conditions. An increased complexity in blood pressure was also found by Voss [36] in patients with dilated cardiomyopathy, observed by Shannon entropy. This specific heart disease and normocapnic hypoxia represent risk states for humans and animals that can be recognized via an increased blood pressure complexity. The nonlinear *PPA *method could support these findings on the basis of increased short-term variability (*SD1*). This index was produced clearer results than linear variability measures, although a high correlation between these indices is given.

According to bivariate analysis using nonlinear methods, findings showed that cardiovascular and respiratory signals from newborn piglets imply not only linear but also nonlinear properties which were altered by the autonomous system as a result of normocapnic hypoxia. Results of *SPPA*, *JSD *and *CCE *analyses are in accordance with findings by Zwiener [5], confirming the existence and alterations of linear and nonlinear properties in cardiovascular and respiratory signals recorded from newborn piglets. Analyses from *JSD *pointed out different results for cardiovascular interactions in contrast to cardio-respiratory interactions. The index *BBI_SYS_JSD*, determining cardiovascular interactions, decreased significantly during prolonged normocapnic hypoxia. This result could be a reference to less vagal activity due to normocapnic hypoxia. Contrastingly, the index *BBI_RESP_JSD*, manifesting the extent of vagal modulation between heart and respiratory rates, showed a continuously decreasing trend during reoxygenation. This index was reduced for all hypoxic and recovery periods, which suggests a high *BBI-RESP *coherence and a pronounced respiratory sinus arrhythmia (*RSA*) after restored oxygen ratio [5].

Significant differences were only affirmed for the cardio-respiratory interaction index during late reoxygenation via multiple testing. Both *JSD *indices led to the assumption that interactions concerning heart rate are more affected by respiration modulation than by blood pressure fluctuations. Presumably, hypoxic exposure is a severe interference to the newborn piglet whose immature autonomous system, especially with regard to respiration regulation, needs time to adapt to recovered oxygen fraction. *SPPA *indices discriminated groups involving both univariate and bivariate analyses. Percentages of data points related to specific columns and rows in the Poincaré plot were thus either significantly increased or decreased during normocapnic hypoxia. One noticeable *SPPA *index was *BBI_DIA_col5*. This index revealed an increase in point percentage caused by hypoxia which showed ratios equal to values for normoxic conditions already in the late hypoxia stage and following periods. The univariate *SPPA *index *RESP_row8 *could contribute to the results of cardio-respiratory analyses in that the *JSD *findings reflect ongoing respiratory regulatory processes other than those found in normoxia. Nonlinear dynamics to investigate the synchronisation of two signals were applied using the *CCE *method, calculating entropies as measures of complexity. Regarding *CCE*, the blood pressure time series revealed a highly significant synchronisation index *SYS_DIA_SI *for HYP1 and HYP2, suggesting a high predictability level for *DIA *when *SYS *is given. The potential reason for the higher blood pressure synchronisation could be the augmented peripheral vasoconstriction, due to increased sympathetic activity during normocapnic hypoxia [34]. After initialising recovery, the synchronisation index decreased in the direction of normoxic values.

A limitation of this study is the small sample number of analysed newborn piglets (N = 7). In experimental animal studies, however, it is usual to investigate such "small-numbered" samples, especially in the case of newborns. Furthermore, the large amount of determined indices (n>100) from uni- and bivariate signal analyses in combination with the small sample size could influence the results of statistical significance tests. Therefore, a statistical adjustment was performed in addition by multiple testing using the Bonferroni-Holm method [30] to confirm the t-test results.

## Conclusions

In conclusion, findings from linear and nonlinear dynamics confirmed the initially higher complexity and higher degree of interactions between cardiovascular and respiratory signals under increased sympathetic influence [2,6,37] due to normocapnic hypoxia. Ongoing normocapnic hypoxia leads to a gradual decrease in cardiovascular variability and complexity. Thus, a moderate adaptation of the autonomous system due to the restricted oxygen supply during late normocapnic hypoxia is already present. During reoxygenation, regulating mechanisms related to ventilation are prolonged and ongoing, presumably due to an unstabilised blood acid-base balance [38-40]. By contrast, the adaptation of *HRV, BPV *and cardiovascular interactions proceed much faster and seem to play a superior role in the autonomous regulatory process. To reveal these findings, it is necessary to analyse more than one short-term section (dynamical behaviour) of non-invasive parameters during prolonged hypoxia and reoxygenation. Furthermore, extensive blood gas analyses could be reduced in the case of non-invasive monitoring of newborns suffering from hypoxia. In a further study, the application of appropriate methods to non-stationary transition phases of this animal experiment will be investigated. Investigations of data from healthy premature infants and from neonates with epilepsy are intended to validate the application of this method to humans. To conclude, the results of this study could contribute to the understanding of the compensatory processes of the newborn autonomous system due to postnatal hypoxia and reoxygenation.

## Abbreviations List

ANS: Autonomic nervous system; BBI: Beat-to-Beat Interval; BPV: Blood Pressure Variability; CCE: Cross Conditional Entropy; DIA: Diastolic blood pressure; ECG: Electrocardiogram; HRV: Heart Rate Variability; JSD: Joint Symbolic Dynamics; NN: Normal-to-Normal beat interval; PPA: Poincaré Plot Analysis; SD: Symbolic Dynamics; SI: Synchronisation Index; Std: Standard deviation; SYS: Systolic blood pressure; RSA: Respiratory Sinus Arrhythmia.

## Competing interests

The authors declare that they have no competing interests.

## Authors' contributions

BW and RB conducted the animal experiment and collected and assembled the data. SS and SR drafted the article and analysed and interpreted the data. ME, KS, HW and RB are well-versed in newborn physiology and participated in manuscript revision. AV conceived the study and critically revised it for significant intellectual content. All authors gave final approval of the version to be published.
